# Potential Biological Markers and Treatment Implications for Binge Eating Disorder and Behavioral Addictions

**DOI:** 10.3390/nu15040827

**Published:** 2023-02-06

**Authors:** Gemma Mestre-Bach, Marc N. Potenza

**Affiliations:** 1 Facultad de Ciencias de la Salud, Universidad Internacional de La Rioja, 26006 Logroño, Spain; 2 Department of Psychiatry, School of Medicine, Yale University, New Haven, CT 06510, USA; 3 Connecticut Mental Health Center, New Haven, CT 06519, USA; 4 Connecticut Council on Problem Gambling, Wethersfield, CT 06109, USA; 5 Wu Tsai Institute, Yale University, New Haven, CT 06510, USA; 6 Yale Child Study Center, School of Medicine, Yale University, New Haven, CT 06510, USA; 7 Department of Neuroscience, School of Medicine, Yale University, New Haven, CT 06510, USA

**Keywords:** reward system, ventral striatum, craving, internet gaming disorder, gambling disorder, binge eating disorder, food addiction

## Abstract

The reward system is highly relevant to behavioral addictions such as gambling disorder (GD), internet gaming disorder (IGD), and food addiction/binge eating disorder (FA/BED). Among other brain regions, the ventral striatum (VS) has been implicated in reward processing. The main objective of the present state-of-the-art review was to explore in depth the specific role of the VS in GD, IGD and FA/BED, understanding it as a possible biomarker of these conditions. Studies analyzing brain changes following interventions for these disorders, and especially those that had explored possible treatment-related changes in VS, are discussed. More evidence is needed on how existing treatments (both pharmacological and psychobehavioral) for behavioral addictions affect the activation of the VS and related circuitry.

## 1. Introduction

The reward system is relevant to addictions, both substance and behavioral. Sex differences across neurobehavioral levels have been reported. It has been suggested that men may be more sensitive to the behavioral relevance (salience) of incentive stimuli, and that women and men show no differences in stimulus valence (e.g., losing versus winning money) [1]. Further, sex differences in motivations for engaging in addictive behaviors (positive reinforcement motivations for men and negative reinforcement motivations for women) may be linked to these neurobehavioral relationships [2,3].

This circuit includes structures such as the ventral tegmental area (VTA; a main brain area involved in the production and projected transmission of dopamine) and the ventral striatum (VS; a VTA projection site) and other areas (i.e., cortical regions including the ventromedial prefrontal cortex (vmPFC) and orbitofrontal cortex (OFC) comprising mesocortical pathways) [4].

**Figure 1:**
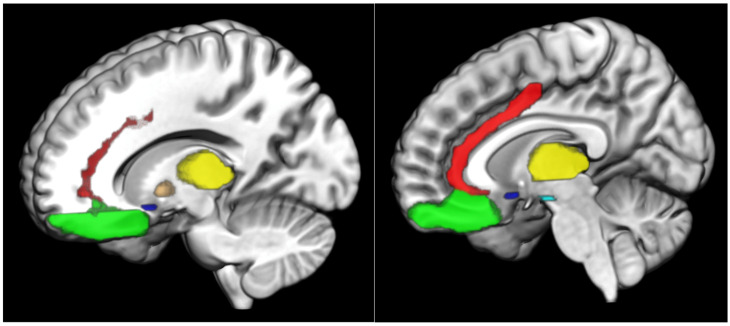
Main brain areas of the reward system anterior cingulate cortex: red; globus pallidus: orange; nucleus accumbens: royal blue; orbitofrontal cortex: neon green; thalamus: yellow; ventral tegmental area: cyan.

The VS encompasses the nucleus accumbens (NAc), together with regions of the medial caudate nucleus and the rostroventral putamen. The VS receives dopaminergic input from the midbrain, as well as cortical input from the anterior cingulate cortex (ACC) and OFC. In turn, the VS projects output to the VTA and the ventral pallidum. These, via the medial dorsal nucleus of the thalamus, project output back to the prefrontal cortex (PFC) [5]. A figure including some of the key reward-related brain regions is included (Figure 1).

The VS, and especially the NAc, contribute importantly to reward processing, being activated by the anticipation and reception of different types of rewards. More specifically, the VS generates predictions about possible gains and establishes a comparison with actual outcomes [6]. In fact, it has been observed that the VS shows a higher activation to positive reward outcomes [7,8,9,10], and this activation seems to decrease if possible losses increase [11]. Moreover, different neurohormones, such as oxytocin, have been linked to reward systems and social behaviors. Oxytocin may influence dopamine and anandamide signaling [12] and relates importantly to sexual arousal and orgasm [13,14] and aspects of maternal bonding [15]. Oxytocin has also been implicated in addictive behaviors and disorders [16,17,18]. Dopamine is a relevant reward-related neurotransmitter [19], and it may influence potentially rewarding behaviors, including food intake [20].

As previously described [21], two complementary theoretical models of addictions associate the roles of the VS with the development and maintenance of addictions: the incentive salience [22] and reward deficiency syndrome theories [23,24]. On the one hand, the incentive salience theory [22] divides motivated behavior into two components: liking (associated with the experienced value of the reward, usually carried by an unconditional stimulus) and wanting (associated with the experienced value of the reward, usually carried by a conditional stimulus/cue). It has been observed that the conditioned cues related to addiction generate increased responses in the VS in individuals with addictions, as well as greater motivated behavior. On the other hand, the reward deficiency syndrome theory [23,24] postulates that individuals with addictions present with alterations in reward pathways and, in particular, a hypoactivation of these brain regions, together with a reduced pleasurable experience derived from non-addiction-related rewards. Therefore, addictive behaviors are used to stimulate the reward circuitry and, consequently, to compensate for reward deficiencies.

In this context, VS-cortical circuitry has been considered relevant to craving [25], and alterations in VS-cortical connectivity have been observed in both substance use disorders (SUDs) [26,27] and behavioral addictions [28,29]. The behavioral addictions most studied at the neurobiological level and accepted in the Diagnostic and Statistical Manual of Mental Disorders, Fifth Edition [30] or the International Classification of Diseases, 11th Revision [31] have been gambling disorder (GD) and internet gaming disorder (IGD; termed gaming disorder in the International Classification of Diseases, 11^th^ Revision). GD is characterized by a maladaptive pattern of gambling behavior that persists despite negative consequences in major areas of life functioning [30]. IGD is characterized by difficulties in controlling excessive/interfering levels of videogaming, often with the presence of tolerance, withdrawal, and negative consequences in major life domains [30]. Likewise, food addiction (FA; typically to highly processed, hyperpalatable and densely caloric foods) has been proposed as another possible addiction, although it has been debated and FA is neither in the Diagnostic and Statistical Manual of Mental Disorders Fifth Edition, nor the International Classification of Diseases, 11th Revision. FA may have overlapping neurobiological systems, particularly when highly palatable foods activate reward circuitry as do substances in SUDs, and these processes may be particularly relevant in people with binge eating disorder (BED) [32]. For example, both in individuals with SUDs and in animal models focused on food intake/binge eating, decreased striatal dopaminergic release has been observed, as well as increased reward thresholds [33]. There is a high co-occurrence of FA with BED and similarities between the two constructs (e.g., although BED has been diagnosed as an eating disorder in the Diagnostic and Statistical Manual of Mental Disorders Fifth Edition [30], it has also been considered an addiction-like behavior [34]); however, they do not completely overlap [35]. Some authors have suggested that individuals with BED who present with FA have greater clinical impairment, possibly due to the impact of an addictive process [35] and, among individuals with BED, those with FA have demonstrated poorer treatment outcomes [36]. Epigenetic mechanisms, including with respect to early life food intake, may contribute to FA and other addictive behaviors [37,38].

The main objective of the present state-of-the-art review was to explore in depth the specific role of the VS in GD, IGD and FA/BED, considering it as a possible biomarker of behavioral addictions with treatment relevance. The aim was to review studies that had analyzed changes at the brain level after the application of interventions for these disorders, and especially those that had explored possible changes in the VS related to these treatments.

## 2. Materials and Methods

The present state-of-the-art review aimed to provide a comprehensive review of the existing literature and to describe the main findings in a narrative format. For this purpose, both PubMed and Google Scholar were searched for scientific articles that had been published in peer-reviewed international journals up to 20 December 2022. Both reviews and original studies with human samples of one or more participants were considered. Articles in both adolescent and adult populations with a diagnosis of GD, IGD or FA/BED were considered. Articles published in both English and Spanish were included. The different searches used terms such as functional magnetic resonance imaging (fMRI), ventral striatum, gambling, gaming, binge eating disorder, food addiction, cognitive behavioral therapy (CBT), treatment, intervention, pharmacotherapy and recovery, among others.

## 3. Results

### 3.1. Ventral Striatal Activation and GD

The VS has been implicated in urges in GD, as well as behavioral and physiological responses to rewards, and it has been hypothesized that differences in VS function may predispose individuals to develop addictions [39]. More specifically, greater gray-matter volume in the right VS has been described in individuals with (versus without) GD [40]. Alterations in dopaminergically innervated regions associated with reward, risk and motivation, such as the VS and vmPFC, have been described in individuals with GD [41,42,43]. Activity in the VS, together with the vmPFC, may reflect both probabilities and magnitudes of potential wins related to risky choices, so that these brain regions could present a coordinated representation of both decisional parameters [44]. However, some authors have been critical of the simplicity of theories of the neurobiological basis of GD, given that both hypersensitivity and hyposensitivity of the VS and other ventral regions of the reward system, such as the vmPFC, have been described [28,45,46,47,48].

Some studies have analyzed the role of VS in the processing of different types of stimuli in individuals with GD. For example, some have observed a blunted activation of the VS and the ventral PFC of individuals with GD in the processing of monetary rewards [49,50,51]. In one study, individuals with (versus without) GD presented differential responses to erotic versus monetary stimuli and, in particular, a reduced sensitivity toward erotic stimuli [52]. Considering the role of VS in instrumental motivation and in the context of imbalance hypotheses, the authors suggest that this asymmetric response pattern may be evidence of a neurophysiological mechanism in which monetary stimuli overpower other types of stimuli in terms of incentive salience. The extent of this differential response between both types of stimuli has been statistically predicted by the severity of GD, so that differential cue reactivity may be a defining feature of GD. Increased VS, dorsolateral prefrontal cortex (DLPFC) and ACC activation have also been described in individuals with GD, compared to controls, when presented with gambling-related cues versus neutral stimuli [53]. Differences in the response to gambling versus food cues have also been explored in individuals with GD. Individuals with (versus without) GD presented greater reactivity to gambling cues, but not to food cues [54]. Likewise, in the GD group, a positive association was observed between gambling-related craving and gambling cue-related activity in the VS, as well as a negative association with functional connectivity (FC) between the VS and the medial PFC [54]. However, other studies have reported relatively blunted VS activation in GD during simulated gambling, with the degree of activation inversely associated with problem gambling severity. Similarly, during the presentation of complex video cues that elicited gambling urges, individuals with (versus without) GD showed blunted VS activation, with similar findings in people with (versus without) cocaine use disorder noted in response to comparable cocaine-related cues. Thus, not all stimuli elicit increased brain responses in the reward-related circuitry in individuals with GD.

Relationships between near-misses (e.g., when 2 reels on a slot machine match but the third does not) and the VS have also been explored. It has been suggested that near-misses activate brain areas similar to wins. In fact, near-misses may lead to greater VS activity compared to non-winning full-miss events [55,56,57]. Neural signaling in the VS (involved in reinforcement learning) may be explained by near-misses being close to wins, so they may be erroneously considered as a sign of skill acquisition and enhance motivations for gambling [58]. This may explain why some studies have observed amplified VS responses to near-misses in individuals with (versus without) GD [58].

#### 3.1.1. Neural Mechanisms of Recovery in GD

##### Pharmacological Interventions for GD

Currently, no pharmacological intervention has been approved by regulatory bodies with an indication for GD, and the study of pharmacotherapies for GD is thus needed. However, the efficacy of different medications for the treatment of GD has been explored, with mixed or negative evidence for lithium, serotonergic antidepressants, catechol-O-methyltransferase inhibitors (e.g., tolcapone), glutamatergic agents, neuroleptics, opioid receptor antagonists and dopamine-1-receptor and dopamine-2-receptor antagonists [59]. The efficacy of these drugs at the neuronal level warrants more examination and, to the best of our knowledge, to date there are no studies that focus on the specific effects of these medications on the VS.

**Bupropion**. Brain measures were obtained from three groups: 15 participants with online GD, 16 participants with IGD and 15 control participants with neither disorder [60]. In the online GD group, after 12 weeks of pharmacotherapy with bupropion, there was a reduction in FC in the default mode network, whereas there was an increase in FC in a cognitive control network. In addition, compared to the IGD group, individuals with online GD showed greater FC in the cognitive control network. Theoretically, pro-dopaminergic effects related to bupropion could stimulate activity of top-down circuitry and impact cognitive control networks, promoting more advantageous decision-making.

**Tolcapone**. A study explored the efficacy of 8 weeks of oral tolcapone, a catechol-O-methyltransferase inhibitor, in 12 individuals with GD through fMRI and an executive-planning task [61]. At baseline, patients with GD, compared to controls, showed fronto-parietal under-activation during the executive-planning task. After pharmacological intervention a reduction of IGD symptomatology was observed, which correlated significantly with the increase in fronto-parietal engagement. Treatment response was also related to a functional allelic variant of the gene coding for the catechol-O-methyltransferase protein, suggesting that personalized medicine approaches may be warranted.

**Fluvoxamine**. A case study explored brain activations with respect to fluvoxamine administration [62]. The individual with GD showed frontal, occipital and parietal activations at baseline with the presentation of playing cards. A decrease in the activated brain areas was observed after fluvoxamine, which was linked to a reduction in the desire to gamble. However, as it is a case report and there was no placebo control, more empirical evidence is needed.

**Lithium.** The efficacy of lithium has been tested in individuals with GD and co-occurring bipolar-spectrum disorders. Positron emission tomography was used to explore mechanisms related to treatment efficacy [63,64]. Lithium may increase the relative glucose metabolic rate in the ventral caudate, albeit not to a statistically significant level, in individuals with GD and bipolar-spectrum disorders. Moreover, lithium may increase the relative glucose metabolic rate in the OFC, DLPFC and posterior cingulate cortex [64].

##### Psychobehavioral Interventions for GD

**Cognitive behavioral therapy (CBT).** A proof-of-concept fMRI study [65] aimed to examine neural mechanisms related to treatment outcomes for GD. Seven treatment-seeking individuals with GD (with co-occurring tobacco use disorder) performed the Stroop task during fMRI before initiating treatment for GD. The treatment consisted of six weeks of CBT, and also included imaginal desensitization motivational interviewing, smoking cessation instruction, and either the amino-acid dietary supplement N-acetyl cysteine or placebo. N-acetyl cysteine is a nutraceutical that can influence VS dopaminergic function through glutamatergic mechanisms. The authors hypothesized that VS activation during the Stroop task would inversely correlate with pretreatment GD severity. It was further hypothesized that VS activity during the pretreatment Stroop task would correlate with improvements in post-treatment GD symptomatology. A positive correlation was found between GD symptomatology and activation of the right VS, including the NAc, thus providing some support for a role for the VS in treatment outcomes for GD.

##### Neuromodulatory Interventions for GD

Repetitive transcranial magnetic stimulation (rTMS) is a neuromodulatory approach to modify brain circuit function, including those related to control over craving [8,9]. However, studies on this non-invasive procedure in GD are relatively scarce. Thus, it may be relevant to focus on neurobiological similarities between gambling urges and drug craving in SUDs to facilitate advances [66,67]. Existing studies in addictions have considered stimulation of the DLPFC [68,69,70,71]. Through its connections with the VS and the VTA, the DLPFC may influence the function of mesostriatal and mesolimbic pathways. Therefore, stimulation of the DLPFC could potentially increase cognitive control over craving [68]. While this may involve dopaminergic circuits, some authors have suggested that stimulation of the DLPFC may have an impact on GABA and glutamate levels in this brain region and connected circuitry, which includes the VS. This may theoretically facilitate dopamine release indirectly in the mesocortical pathway, reducing levels of craving, impulsivity and reward-seeking [72].

### 3.2. Ventral Striatal Activation and IGD

A tripartite neurocognitive model has been proposed for IGD [73]. This would include a dual process that suggests that both the development and maintenance of this disorder may involve hyperactivity of the reward system (amygdala-VS-dependent) in response to gaming-related cues, together with the weakening of inhibition (DLPFC-dependent). The third system could refer to the interoceptive awareness system (insula-dependent), which may involve converting somatic signals of reward obtained with video games into subjective desire. Based on this theoretical model, it was hypothesized that IGD severity scores among people who played *League of Legends* would be positively related to the activation of the reward system and that, in turn, they would be negatively linked to the activation of prefrontal areas [74]. It was observed that the VS was more strongly activated in people who played *League of Legends* in response to gaming-related cues, and that the left frontal pole and DLPFC were more weakly activated compared to comparison subjects (who did not play *League of Legends*). Stronger activations of the VS have been reported in individuals with (versus without) IGD in other studies [75,76], as well as less activation in the DLPFC and inferior parietal lobe during the evaluation of potential losses and the risk perception [76]. This increased activation of the VS in the face of addiction-related cues appears to be a shared factor between IGD, GD and SUDs [77].

Data provide insights into the negative associations between craving and cue-induced activations in the VS [78], as well as the positive relationship between both factors in the dorsal striatum [79,80] in the case of individuals with SUDs. In this context, in individuals with IGD, brain activity in the left VS was inversely related to gaming-cue induced craving, which may suggest that in IGD there is a decreased involvement of the VS in cue processing [81]. A positive association between activation of the dorsal striatum and IGD duration was noted. In individuals with IGD, a negative association was observed between right VS volumes and cue-induced activity in the left putamen. Abnormal resting-state FC within a dorsal striatum network and VS network (i.e., reduced caudate–DLPFC and NAc–OFC resting-state FC strength) in individuals with IGD has also been described [82]. It has been suggested that the VS has an essential role in the early stages of addictions, while in later stages the dorsal striatum may be more involved in the compulsive aspects of addictions, highlighting a relevant shift [83,84].

The frequent use of video games has been associated with dopamine release in the VS [85], as well as with an increased volume of the left VS [86], which may reflect an alteration in reward processing [87]. Similarly, in individuals with IGD, larger volumes of the VS, specifically the NAc, as well as the dorsal striatum, specifically the caudate, have been observed [88]. However, other studies have obtained opposite results, suggesting that individuals with (versus without) IGD may have reduced striatal volumes, especially in the VS [89,90].

Some studies have highlighted an association between the VS and the left OFC (associated with cue reactivity in individuals with IGD) and the right inferior frontal gyrus (associated with inhibition processing) in individuals with IGD [91]. Other studies have found that, in the VS, individuals with IGD, compared to those with recreational game use, exhibited lower FC with the middle frontal gyrus, especially on the left and mostly in the supplementary motor area (involved in motor planning and execution) [92]. Therefore, considering that the VS is associated with the learning values of stimuli, the lower FC with the middle frontal gyrus may suggest that individuals with IGD present a possible disconnection between stimulus evaluation and behavioral responses in domains such as response inhibition [92]. Likewise, during response inhibition under high-load tasks, individuals with IGD present an increased inefficient engagement of the VS and DLPFC, which may highlight their vulnerability to inappropriate response inhibition with higher-level cognitive skills [93].

Taking into account specific aspects of IGD, it has been suggested that greater IGD severity may increase the effective connectivity between the VS bilaterally [94]. IGD severity has also been associated with VS bias for monetary rewards [95], and has been negatively associated with FC between frontal and striatal brain areas [96]. Furthermore, the number of IGD symptoms appears to be negatively associated with dorsal-ACC-VS resting-state FC, suggesting that it may be considered as a biomarker of IGD, as well as an important target for interventions addressing this disorder [97]. The functional coupling between dorsal ACC and VS may be involved in feedback learning. Therefore, the alterations presented by individuals with IGD may imply difficulties in representing value signals attached to action outcome relationships and, consequently, learning problems [98].

#### 3.2.1. Neural Mechanisms of Recovery in IGD

Several studies have explored neural mechanisms involved in the recovery of individuals with IGD. For example, Dong et al. [99] hypothesized that brain regions involved in craving, such as the striatum, may show less activation in those individuals recovered from IGD (without formal intervention), compared to those with active IGD, when exposed to gaming cues. The authors observed decreased craving responses to gaming-related cues at both subjective and neural levels in individuals with IGD in recovery. More specifically, individuals recovered from IGD showed relatively diminished AAC activation and decreased gaming-cue related activations in the vmPFC/OFC and lentiform nucleus, thus possibly presenting a lower motivation to perform gaming. Likewise, cue-elicited activation of the lentiform nucleus has also been related to the development of IGD in individuals with regular videogame use [100]. Therefore, in both the emergence and recovery of IGD, gaming-cue elicited lentiform activity should be considered. Therefore, these authors highlighted the need to consider craving reduction in IGD as a potential neural target for interventions such as neuromodulation or behavioral approaches [99].

##### Pharmacological Interventions for IGD

Several pharmacological options have been suggested for IGD. Drugs used for treating depression (bupropion and escitalopram) and attention deficit/hyperactivity disorder (methylphenidate and atomoxetine), conditions that frequently co-occur with IGD, have been evaluated in IGD treatment [60,101,102,103,104]. However, no pharmacological intervention has a formal indication for the treatment of IGD, and none of the proposed options have been sufficiently evaluated for their efficacy and tolerability [105].

Few studies have explored the neural effects of pharmacological interventions in individuals with IGD, and of these, to the best of our knowledge, none have specifically examined the effect on the VS. In one study of IGD, GD and control comparison subjects [60], a reduction in FC in the posterior default mode network and between the posterior default mode network and the cognitive control network was observed in individuals with IGD after 12 weeks of bupropion treatment. Furthermore, the authors noted that FC in the default mode network was positively correlated with changes in IGD symptomatology after pharmacological intervention. A 12-week double blind prospective trial compared bupropion and escitalopram in 30 individuals with IGD and major depressive disorder (15 in each group) [106]. In the case of bupropion, a significant reduction in FC was observed in the salience network and between the salience network and the default mode network. In contrast, in the escitalopram group, only a reduction of FC in the default mode network was found. Speculatively, bupropion may show greater efficiency than escitalopram in reducing impulsivity and attentional symptoms, while both drugs may reduce depressive and IGD symptoms.

##### Psychobehavioral Interventions for IGD

Following behavioral interventions in patients with addictions (both substance and behavioral), changes in cortico-striatal function have been observed. In studies of individuals with SUDs, cortico-striatal circuitry has been a proposed mechanism underlying different treatments, such as cognitive therapy [107], mindfulness-oriented recovery enhancement [108], and cue-exposure based extinction training [109]. This may imply that cortico-striatal circuitry may also be altered in IGD, and the improvement of the functionality of this circuitry may be a mechanism underlying psychobehavioral interventions.

**CBT**. Han et al. [110] compared 26 individuals with IGD and 30 comparison subjects without, and examined the efficacy of CBT in 20 individuals with IGD. The CBT consisted of 12 group sessions lasting 1.5–2 h each in which topics such as emotion recognition, impulse control, and recognition and control of addictive behaviors were addressed. The authors hypothesized that individuals with IGD would present abnormal brain connectivity in prefrontal-striatal areas and that CBT would be effective in regulating this abnormal function. The results of the study suggested that CBT may regulate the abnormal low-frequency fluctuations in prefrontal-striatal areas of individuals with IGD and, consequently, improve IGD symptomatology.

The efficacy of CBT was contrasted with the efficacy of virtual reality in a study involving 36 individuals with IGD, 24 of whom were randomly assigned to the CBT group, while the remaining 12 were assigned to the virtual reality group [111]. The virtual reality program, lasting eight sessions, was designed to increase balanced activation of the brain reward circuitry with stimulation of the limbic system. More specifically, the intervention aimed to stimulate both the striatum (linked to craving) and the temporal lobe (linked to aversion), as well as to facilitate limbic-regulated responses to reward stimuli. The process was tested by pairing scenes of aversive consequences of gaming behavior and craving-inducing game-related stimuli. Both CBT and the virtual reality program showed similar effects on the reduction of IGD symptomatology. Likewise, the virtual reality program improved balance in the cortico-striatal-limbic circuit. Therefore, the authors suggested that virtual reality may be an effective tool for the facilitation of limbic-regulated responses to rewarding stimuli. Studies exploring the specific effects of CBT on VS activity are needed.

**Family therapy**. Han et al. [112] observed a change of striatal activity after family therapy in adolescents with IGD. The family therapy consisted of five sessions to increase family cohesion, along with two assessment sessions. Those adolescents with IGD and a poor family relationship may use video games as a compensatory strategy for reward deficits (potentially related to VS activity) relating to poor parental support. Both romantic and maternal love may influence activation of the striatum, as part of reward circuitry.

**Craving behavioral intervention (CBI)**. Several studies have explored the impact of CBI on the VS of individuals with IGD. CBI is an intervention focused on reducing craving levels. The different studies that have used this intervention have used it in a group format (between 8–9 individuals) and organized in 6 weekly sessions in which topics such as perception and recognition of craving and training in coping skills and mindfulness to reduce craving are addressed [113].

Zhang et al. [113] evaluated effects of CBI on the resting-state FC of the VS. More specifically, the authors compared 76 individuals with IGD with 41 control participants, assessing resting-state FC of the VS. Of the individuals with IGD, 25 received CBI while 19 did not. Individuals with (versus without) IGD showed a significantly higher resting-state FC of VS to the left middle frontal gyrus, the left inferior parietal lobule and the right inferior frontal gyrus. When comparing the posttest with the pretest of IGD individuals who had received CBI, a significant reduction of the strength of VS-left inferior parietal lobule connectivity was observed (*p* = 0.001). However, the group of IGD individuals who had not undergone CBI showed no change between pretest and posttest (*p* = 0.73). In the light of these findings, the authors suggested that VS-left inferior parietal lobule FC may be considered as a stable biomarker for CBI efficacy in IGD. However, VS-left middle frontal gyrus, and especially VS-right inferior frontal gyrus connectivity, may not be regarded as specific markers of CBI efficacy, since they decreased significantly in both groups (the one that had undergone CBI and the one that had not). Considering this potential biomarker, non-invasive techniques, such as transcranial magnetic stimulation and transcranial direct current stimulation (tDCS) may be considered for treatment of IGD.

In a subsequent publication, Zhang et al. [114] hypothesized that individuals with (versus without) IGD may have greater activation of reward-related areas, including the VS, involved in cue-induced craving. The authors analyzed the efficacy of CBI by comparing a group of 23 individuals with IGD who received the intervention with a group of 17 individuals with IGD who did not. At a behavioral level, CBI reduced cue-induced craving and IGD severity. However, the intervention, at the neural level, did not “normalize” IGD-related cue-induced brain activation identified at baseline. Therefore, CBI may not have impacted the reward system and, specifically, the VS. However, CBI appeared to have impacted another brain region, the anterior insula, which at baseline had shown no differences between individuals with and without IGD. Therefore, the authors suggested that this intervention, rather than directly altering regions involved in reinforcement, may modulate brain areas involved in higher-level cognitive functioning. Thus, it was suggested that future studies may test combinations of CBI with other interventions that have a direct effect on the VS and other regions involved in cue reactivity, such as pharmacological interventions, non-invasive procedures such as transcranial magnetic stimulation or invasive procedures such as deep-brain stimulation.

Wang et al. [115], who also administered CBI to individuals with IGD, observed that some reward-related brain areas, especially the ventral and dorsal striatum, were not involved in their classification analysis. These findings are consistent with previous studies, which found that these specific areas did not show differences in activation to cue reactivity tasks [116]. One explanation suggested by the authors is that the individuals included in their study were in later stages of the addiction process, so it is possible that videogame-related stimuli may be evoking less robust responses than the gaming behavior itself.

Finally, Liu et al. [117] observed an association between changes in craving levels and IGD severity at six months after CBI and connectivity differences in the left angular gyrus and vmPFC. However, given that most previous studies have focused on the VS and other regions involved in reward processing, such as the vmPFC, the specific role of the angular gyrus in craving warrants direct examination.

**Equine-assisted therapy**. Kang et al. [118] explored the neural correlates of equine-assisted therapy and insecure attachment in 15 adolescents with and 15 without IGD. This intervention included 12 x 60-min therapeutic riding sessions. Although the authors did not specifically focus on examining the VS, they observed that the intervention reduced IGD severity and increased FC in an affective network, which was associated with attachment in both adolescents with and without IGD.

##### Neuromodulatory Interventions for IGD

Some studies have used transcranial magnetic stimulation in individuals with IGD [119,120,121]. Most of these studies, as with GD, have focused on the DLPFC. Cue-induced craving may involve automatic responses to addiction-related cues that may be difficult to attenuate [120]. TDCS of the DLPFC may enhance control over both negative emotions and craving [121] and have effects on control and reward systems [120]. Hyperactivation of reward networks (including the striatum) may induce neuroadaptations in craving, in response to addiction-related stimuli [120]. Therefore, weakening the neural-processes related behaviors linked to craving may require improving executive control in individuals with IGD [120]. RTMS may also augment activity in frontostriatal circuits and, consequently, reduce craving and improve cognitive functioning [122].

### 3.3. Ventral Striatal Activation and BED/FA

FA models are based in part on neurobiological evidence in individuals with obesity, although this approach has been debated. Individuals with obesity may demonstrate greater cue-evoked activation of the VS and other cortical-striatal areas that encode food-related reward cues [123,124,125]. Furthermore, this food-cue evoked activation has been associated with subjective assessments of craving [126,127]. However, the specific role of the VS in individuals with FA warrants further consideration. FA has shown similarities with SUDs with respect to ventral striatal sensitization, but arguably not dorsal striatal alterations [128]. FA scores have been associated with VS activity [129]. Fasting has been associated with greater sensitivity of VS to the reward value of food, and this relationship may be modulated by FA [128]. At a theoretical level, FA has been associated with a reduced responsivity of a dorsal striatum network to changes in the reward value of food following satiety [130,131]. Similarly, individuals with BED may present a greater sensitivity to rewarding stimuli, associated with increased activity in the VS and other reward-related brain regions, such as the insula or the OFC, during the presentation of food cues [132].

#### 3.3.1. Neural Mechanisms of Recovery in BED/FA

##### Pharmacological Interventions for BED/FA

As described previously [133], pharmacological treatments have been examined for BED including monoamine stimulants; monoamine reuptake inhibitors; 5-HT2C and trace amine receptor agonists; mu opioid, NOP, orexin 1, cannabinoid and receptor antagonists; glutamate N-methyl-D-aspartate receptor antagonists; Sigma_1_ ligands; and GABA_B_ receptor agonists. However, although the efficacy of these options has been explored to a greater or lesser extent, there is an evident lack of studies examining the specific impact of these drugs on the VS.

**Sibutramine**. Few studies have tested the efficacy of pharmacological interventions at a neurobiological level. Balodis et al. [134] tested 19 patients with obesity and BED over 4 months of treatment with sibutramine and cognitive behavioral-self-help interventions, alone or in combination. Together with findings from their previous study [135], the authors suggested that individuals with BED have relatively diminished activation of reward circuitry, including the VS, to monetary cues. Further, among individuals with BED, those who exhibited persistent bingeing behaviors following treatment demonstrated less activation of reward circuitry (including the VA) to monetary cues at baseline. The reduced frontostriatal responses to non-food rewards seem to be relevant to treatment outcome, which suggest a certain pathophysiology of BED [133] that is similar to those of SUDs and their treatment responses, including in tobacco and cocaine use disorders.

**Lisdexamfetamine**. The effects of lisdexamfetamine at the neurobiological level have also been tested in individuals with BED [136]. Fleck et al. [137] observed that, at baseline, 20 women with BED had greater activation of the striatum, ventrolateral PFC and globus pallidus during the presentation of food images. This activation was related to treatment outcome. After 12 weeks of treatment, women with BED showed significant reductions in globus pallidus activation. Likewise, reductions in vmPFC correlated with reductions in binge eating behaviors. Other studies have associated lisdexamfetamine with reduced activity bilaterally in the thalamus in individuals with BED when viewing food pictures [138]. Likewise, lisdexamfetamine appears to reduce motor impulsivity, but does not appear to have an effect on working memory or emotional bias [138]. Lisdexamfetamine is currently the only drug with formal regulatory approval for treating BED.

**Mu opioid receptor antagonist**. The effects of GSK1521498, a mu opioid receptor antagonist, have also been tested in individuals with BED [139]. Twenty-eight days of treatment were associated with a significant reduction in pallidatum/putamen responses to high-calorie food images. However, subjective liking toward these images increased after pharmacological treatment.

##### Psychobehavioral Interventions for BED/FA

**Reward re-training (RTT)**. The efficacy of RTT has been explored at a neurobiological level. RRT is a 10-session group-based behavioral treatment that aims to augment standard CBT [140]. It focuses on the identification and implementation of activities to increase responses to monetary and sustained rewards. RRT appears to have an impact on the hypo- and hyper-reward response of individuals with BED, as assessed by self-report and fMRI [140]. However, empirical evidence about the specific impact of different psychobehavioral interventions on the VS in individuals with BED/FA is needed.

**CBT**. Thirty-five studies that have used CBT to treat individuals with BED were identified in a meta-analysis [141]. However, there is a lack of studies assessing CBT in relation to the reward system. To the best of our knowledge, only the study by Balodis et al. [134] (mentioned above) examined brain relationships with multiple treatments including CBT. More research is needed to examine brain measures related to CBT specifically and its putative active ingredients, as is the case for other disorders like GD [65].

##### Neuromodulatory Interventions for BED/FA

A proof-of-concept study examined tDCS in individuals with BED (Burgess et al., 2016). Thirty individuals with BED were administered tDCS to DLPFC areas. However, the mechanisms of tDCS on the DLPFC in BED are unclear. On the one hand, stimulation may disrupt reward neurocircuitry from signaling. On the other hand, it may accelerate satiety signaling, thereby decreasing food consumption. These findings suggested different recovery processes in BED.

Dunlop et al. [142] administered 20–30 sessions of rTMS in 28 subjects with anorexia nervosa, binge-purge subtype or bulimia nervosa. Individuals were stratified into responder and non-responder groups regarding ≥50% reduction in weekly binge/purge frequency. Enhanced frontostriatal connectivity was linked in responders to dmPFC-repetitive TMS for binge/purge behaviors. In the non-responder group, rTMS generated paradoxical suppression of frontostriatal connectivity. However, studies exploring effects of non-invasive procedures on the VS in individuals with BED/FA are needed.

## 4. Limitations and Future Studies

First, it should be noted that the narrative nature of the present state-of-the art review has potential biases that future studies could resolve by conducting systematic reviews following PRISMA standards. Second, few studies have evaluated possible biomarkers in behavioral addictions and the effect of treatments on them. Even fewer studies have focused on the impact of both pharmacological and psychobehavioral interventions on the VS and the reward system. Moreover, studies that currently exist have typically used very small samples and heterogeneous treatments, making the results difficult to generalize and compare. Finally, the lack of clinical recognition of an FA construct makes its evaluation and treatment difficult and promotes heterogeneous study methodologies. Future studies could explore the specific role of the VS in behavioral addictions and the impacts of different treatments on them using large samples and less biased study designs.

## 5. Conclusions

The VS has been suggested as a relevant biomarker in behavioral addictions including GD, IGD and FA/BED due to its role in reward processing. However, more evidence is needed on how existing treatments for these behavioral addictions (pharmacological, psychobehavioral and neuromodulatory) may impact the activation of this specific brain area and its connectivity with others.

## Data Availability

Not applicable.

## Glossary

ACC	anterior cingulate cortex
BED	binge eating disorder
CBT	cognitive behavioral therapy
CBI	craving behavioral intervention
DLPFC	dorsolateral prefrontal cortex
FA	food addiction
FC	functional connectivity
fMRI	functional magnetic resonance imaging
GD	gambling disorder
IGD	internet gaming disorder
NAc	nucleus accumbens
OFC	orbitofrontal cortex
PFC	prefrontal cortex
rTMS	repetitive transcranial magnetic stimulation
SUDs	substance use disorders
tDCS	transcranial direct current stimulation
VS	ventral striatum
VTA	ventral tegmental area
vmPFC	ventromedial prefrontal cortex
